# Supplementary material for: Acoustic non-Hermitian skin effect from twisted winding topology
Source: Nat Commun. 2021 Nov 2;12:6297. doi: 10.1038/s41467-021-26619-8 (PMC8563885; doi:10.1038/s41467-021-26619-8)
Supplement: Supplementary file 1 — Supplementary Information [file 41467_2021_26619_MOESM1_ESM.pdf]

Supplementary Information

Acoustic non-Hermitian skin effect from twisted winding topology

Li Zhang[1,2,#], Yihao Yang[1,2,#*], Yong Ge[3,#], Yi-jun Guan[3], Qiaolu Chen[1,2], Qinghui Yan[1,2], Fujia Chen[1,2], Rui Xi[1,2], Yuanzhen Li[1,2], Ding Jia[3], Shou-qi Yuan[3], Hong-xiang Sun[3,*], Hongsheng Chen[1,2,*] and Baile Zhang[4,5,*]

[1]Interdisciplinary Center for Quantum Information, State Key Laboratory of Modern Optical Instrumentation, College of Information Science and Electronic Engineering, Zhejiang University, Hangzhou 310027, China.

[2]ZJU-Hangzhou Global Science and Technology Innovation Center, Key Lab. of Advanced Micro/Nano Electronic Devices & Smart Systems of Zhejiang, ZJU-UIUC Institute, Zhejiang University, Hangzhou 310027, China.

[3]Research Center of Fluid Machinery Engineering and Technology, School of Physics and Electronic Engineering, Jiangsu University, Zhenjiang 212013, China.

[4]Division of Physics and Applied Physics, School of Physical and Mathematical Sciences, Nanyang Technological University, 21 Nanyang Link, Singapore 637371, Singapore.

[5]Centre for Disruptive Photonic Technologies, The Photonics Institute, Nanyang Technological University, 50 Nanyang Avenue, Singapore 639798, Singapore.

[#] These authors contributed equally

[*]*yangyihao@zju.edu.cn* (Yihao Yang); *jsdxshx@ujs.edu.cn* (Hong-xiang Sun); *hansomchen@zju.edu.cn* (Hongsheng Chen); *blzhang@ntu.edu.sg* (Baile Zhang)

Supplementary Note 1: More complex unconventional topological windings

Our acoustic platform can generate more complex unconventional topological windings, as the nonreciprocity can be applied between arbitrary two sites. Here, we present two models. As shown in Fig. S1(a), the first example has reciprocal nearest-neighbor coupling $\kappa_1 = 6$ Hz (yellow line) and unidirectional next-nearest-neighbor coupling $\tilde{\kappa}_a = -11 + 3.9i$ Hz (blue line). The corresponding lattice Hamiltonian is $H = \kappa_1 e^{ika} + \kappa_1 e^{-ika} + \tilde{\kappa}_a e^{-2ika} + \omega_0 - i\gamma_0$. This model is similar to the counterpart in Fig. 4b, except for the strength of the reciprocal coupling that can be tuned by changing the cross-linked waveguide sizes. One can see that the complex energy plane is divided into four parts, including three regions with $v = -1$ (yellow shading) and one region with $v = -2$ (Fig. S1b).

As presented in Fig. S1c, the second model has reciprocal nearest-neighbour coupling κ_1 (yellow line), forward positive coupling $\tilde{\kappa}_a$ (blue line), and backward negative coupling $-\tilde{\kappa}_a$ (red line). The unidirectional coupling sign can be modified by adding a π phase shifter to the active component. Here, we set $\kappa_1 = 24$ Hz and $\tilde{\kappa}_a = -11 + 3.9i$ Hz. The extracted complex energy spectrum encloses four areas with a winding number $v = -1, +1, -1, +1$ from left to right, respectively, as shown in Fig. S1d.

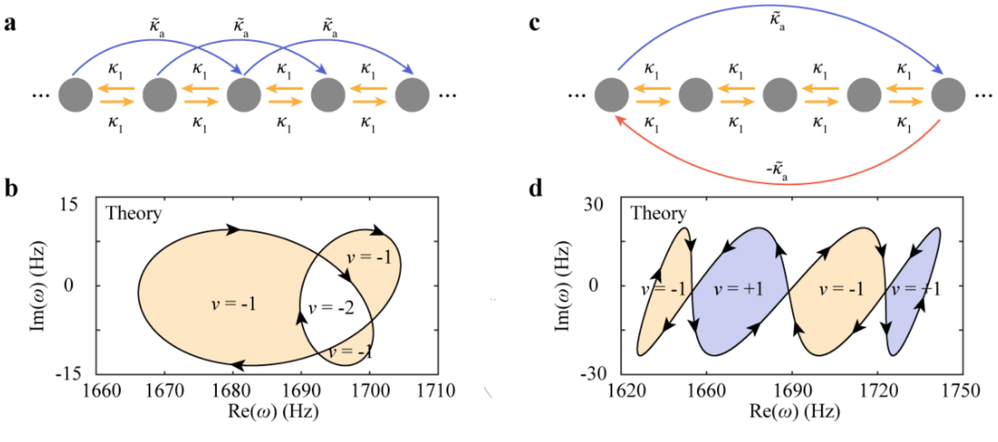

Supplementary Fig. 1 More complex unconventional spectral windings. a Schematic of the non-Hermitian model 1. The yellow lines and blue lines denote the reciprocal and non-reciprocal next-nearest neighbour couplings, respectively. **b** Analytically-calculated complex energy spectrum. Light yellow shading denotes winding number $v = -1$ and the enclosed region implies winding number $v = -2$. **c** Schematic of the non-Hermitian model 2. The yellow

lines and blue/red lines denote the reciprocal and unidirectional couplings, respectively. **d** Analytically-calculated complex energy spectrum. Light yellow (blue) shading denotes a winding number v = -1(+1).

Supplementary Note 2: Details of the two coupled acoustic cavities

Figure S2 displays the model of the two coupled acoustic resonators. Each cavity has length l = 9.2 cm, width w = 7.2 cm, and height h = 11.2 cm. The distance between two resonators is s = 2.4 cm, and two waveguides have the same width d = 3.4 cm.

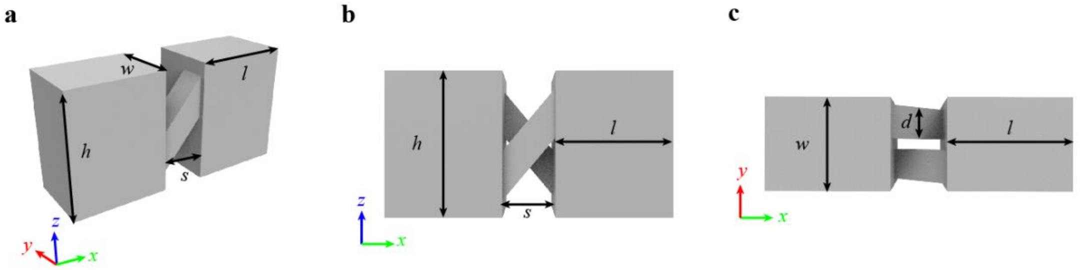

Supplementary Fig. 2 Details of the two coupled acoustic cavities. a 3D view. **b** Front view. **c** Top view.

Supplementary Note 3: Coupled-mode theory (CMT) for two-resonator model

As shown in Fig. 2(a) in the main text, the two-resonator model is composed of two acoustic cavities connected via dual crossed waveguides, an active component amplifying the sound from cavity 1 to cavity 2 unidirectionally, a source, and a detector. As shown in Fig. S3(a), the two cavities have the same resonance frequency ω_0 and intrinsic loss γ_0, and two crossed waveguides provide the reciprocal coupling coefficient κ_1. The source and the detector are inserted into the cavities with the same coupling strength γ_1 (considering the source tube and the detector tube have the same size). The active component provides complex unidirectional coupling $\tilde{\kappa}_a$. According to the CMT[1], when the wave is incident to cavity 1 (Fig. S3), the dynamic equation can be described as

$$\frac{d}{dt}\begin{bmatrix} a_1 \\ a_2 \end{bmatrix} = \begin{bmatrix} -i\omega_0 - \gamma_1 - \gamma_0 & -i\kappa_1 \\ -i(\kappa_1 + \tilde{\kappa}_a) & -i\omega_0 - \gamma_1 - \gamma_0 \end{bmatrix} \begin{bmatrix} a_1 \\ a_2 \end{bmatrix} + \begin{bmatrix} \sqrt{2\gamma_1} \\ 0 \end{bmatrix} s_{1+} \quad , \tag{S1}$$

where a_1 (a_2) is the mode in cavity 1 (2), s_{1+} represents the incident wave from cavity 1. For the case

when the wave is incident to cavity 2, change $\begin{bmatrix}\sqrt{2\gamma_1}\\0\end{bmatrix}s_{1+}$ to $\begin{bmatrix}0\\\sqrt{2\gamma_1}\end{bmatrix}s_{2+}$, where s_{2+} represents the wave incident to cavity 2.

The corresponding effective Hamiltonian is,

$$H=\begin{bmatrix}\omega_0-i(\gamma_0+\gamma_1) & \kappa_1\\ \kappa_1+\tilde{\kappa}_a & \omega_0-i(\gamma_0+\gamma_1)\end{bmatrix}. \tag{S2}$$

It can be seen that in this Hamiltonian, the Hermiticity, time-reversal symmetry, and reciprocity are broken simultaneously[2].

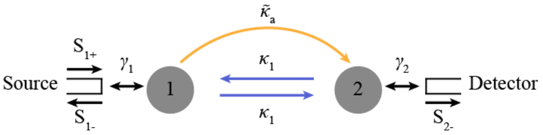

Supplementary Fig. 3 The CMT model for the two-resonator model. The CMT model for the two-resonator model composed of two acoustic cavities.

Supplementary Note 4: Transmission coefficients for the two-resonator mode

According to Eq. 1, the transmission coefficients are

$$s_{21}=-i\frac{2\gamma_1(\kappa_1+\tilde{\kappa}_a)}{\left[i(\omega-\omega_0)-\gamma_1-\gamma_0\right]\left[i(\omega-\omega_0)-\gamma_1-\gamma_0\right]+(\kappa_1+\tilde{\kappa}_a)\kappa_1}, \tag{S3}$$

$$s_{12}=-i\frac{2\gamma_1\kappa_1}{\left[i(\omega-\omega_0)-\gamma_1-\gamma_0\right]\left[i(\omega-\omega_0)-\gamma_1-\gamma_0\right]+(\kappa_1+\tilde{\kappa}_a)\kappa_1}. \tag{S4}$$

In the reciprocal model without the unidirectional coupling, the transmission coefficients are

$$s_{21}=s_{12}=-i\frac{2\gamma_1\kappa_1}{\left[i(\omega-\omega_0)-\gamma_1-\gamma_0\right]\left[i(\omega-\omega_0)-\gamma_1-\gamma_0\right]+\kappa_1^2}. \tag{S5}$$

We use Eq. (S3), Eq. (S4) and Eq. (S5) to fit the three measured transmission spectra and retrieve the resonance frequency ω_0 = 1706 Hz, the reciprocal coupling κ_1= 24 Hz, the unidirectional coupling $\tilde{\kappa}_a$ = -11 + 3.9i Hz, the intrinsic loss γ_0 = 2.13 Hz, and the coupling strength γ_1=1.77 Hz.

Supplementary Note 5: Active acoustic component

Figure S4a displays a photograph of the active component, where the standard non-inverting amplifier and the electrical circuit are fabricated with the printed circuit board (PCB) technique. The active amplifier component consists of a power amplifier and two highpass filters, as depicted in Fig. S4b. The input and output signal has a phase difference due to the resistances and capacitances in the amplifier and the filters.

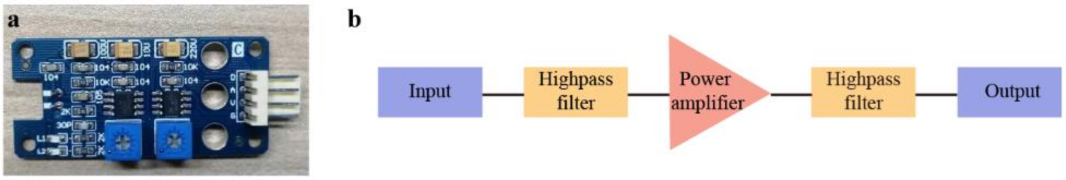

Supplementary Fig. 4 Active acoustic component. a Photograph of the active component. **b** Schematic of the active component.

Supplementary Note 6: Transmission measurement of a pulse in the time domain in the acoustic crystal with the nearest-neighbor non-reciprocal coupling

We launch an acoustic pulse, ranging from 1680 to 1720 Hz, from site 1 and measure the transmission at each site. As depicted in Fig. S5, it can be seen that the transmission toward the right boundary (site 20) decreases dramatically as the site number increases.

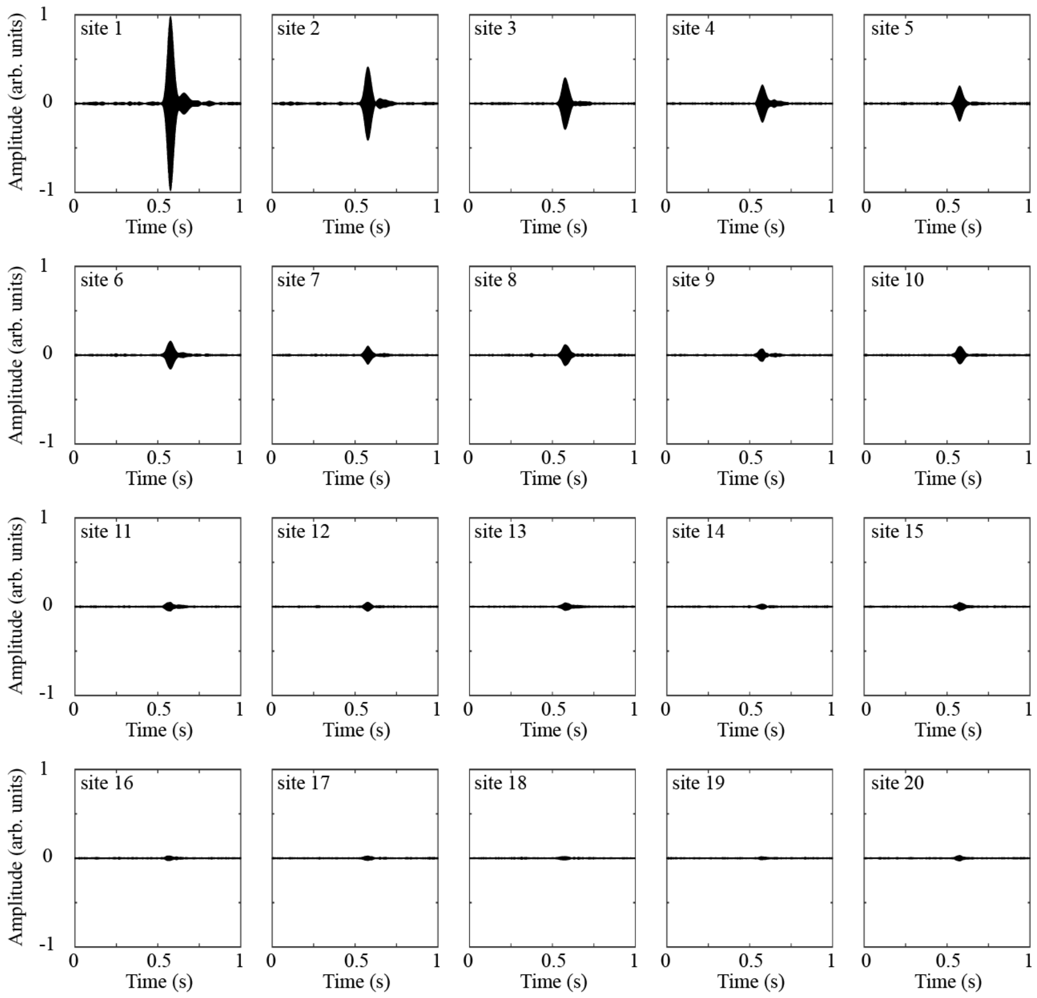

Supplementary Fig. 5 Transmission measurement of a pulse in the time domain. Transmission measurement of a pulse in the time domain from site 1 to site 20 in the acoustic crystal with the nearest-neighbor non-reciprocal coupling. The measured results are normalized by the amplitude at site 1.

Supplementary Note 7: Decrease in amplitude and decay ratio

In Fig. 3e, a Gaussian-like pulse, covering the frequency range from 1680 to 1720 Hz, is launched from site 1 and detected at site 20. The transmission from the left boundary towards the right boundary almost vanishes. In order to compare the decrease in the amplitude, we apply the Fourier transform to obtain the corresponding frequency spectra [Fig. S6(a) and S6(b)]. The decrease in the amplitude is defined as the difference between the amplitude of the input pulse and that of the detected pulse, which is shown in Fig. S6(c). In addition, the decay ratio defined as the

ratio between the decrease in the amplitude and amplitude of the input pulse is also plotted in Fig. S6(d). In this case, the energy is pumped out of the system.

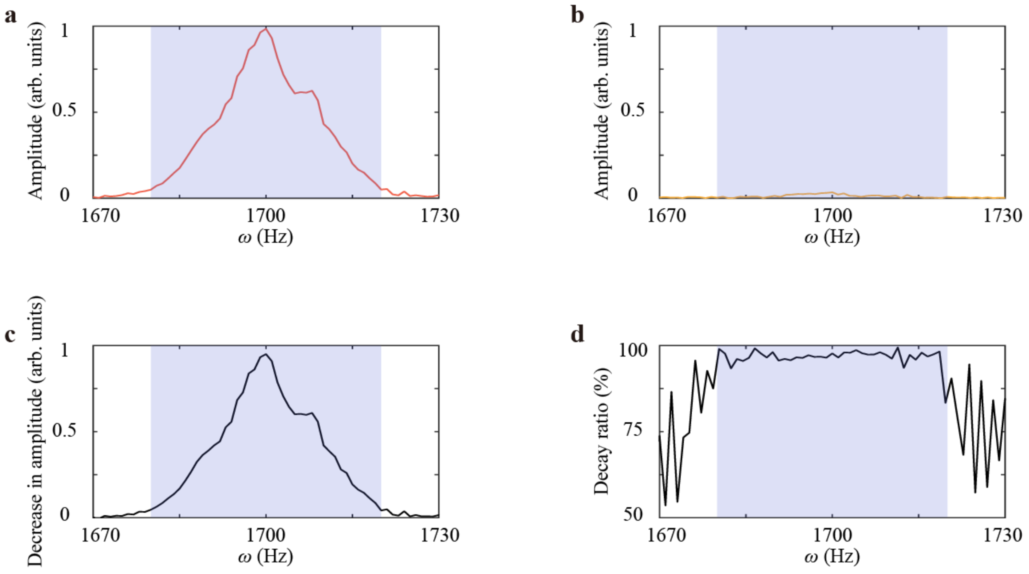

Supplementary Fig. 6 Decrease in the amplitude and the decay ratio of a pulse in the time domain. a, **b** Frequency spectra obtained by applying Fourier transform to the time-domain signal at site 1 and site 20, respectively. The measured results are normalized by the amplitude at site 1. **c** Measured decrease in amplitude as a function of frequency. The measured results are normalized by the amplitude at site 1. **d** Measured decay ratio as a function of frequency. The blue region represents three standard deviations of the Gaussian-like pulse, which covers a frequency range from 1680 to 1720 Hz.

Supplementary Note 8: Measured field intensity distributions in the acoustic crystal with the nearest-neighbor non-reciprocal coupling

In this section, more measured field profiles in the acoustic crystal with the nearest-neighbor non-reciprocal coupling at different frequencies are presented in Fig. S7. It is obvious that the field distribution localized at the left boundary exhibits stronger amplitude, when the source is placed further away from the left.

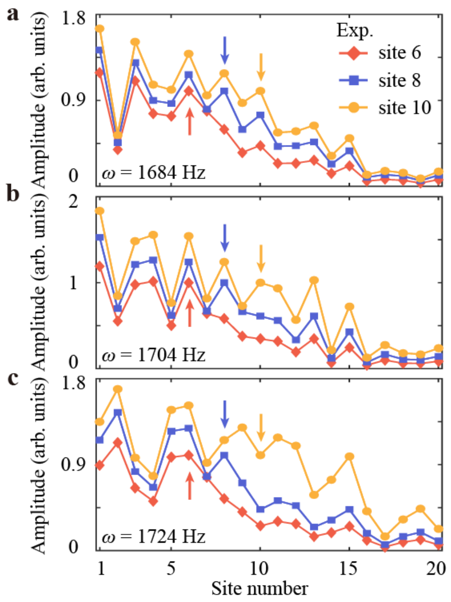

Supplementary Fig. 7 Measured field intensity distributions in the acoustic crystal with the nearest-neighbor non-reciprocal coupling. a-c Field distribution at frequency ω = 1684, 1704 and 1724 Hz, when the source is located at site 6, 8, and 10, respectively. The measured results are normalized by the amplitude at the source location (indicated by little arrows).

Supplementary Note 9 Experimental results of the reciprocal acoustic crystal

To probe the field distribution, we place the source at site 10 and detect the response at each site of the chain (the same sample shown in Fig. 3(a) and the active components are not in operation). As shown in Fig. S8(a), the wave is concentrated on the input site 10 and propagates toward both directions with almost identical amplitude at ω = 1713 Hz. By applying Fourier transform to the measured field distributions in the chain, we obtain the dispersion, as depicted in Fig. S8(b).

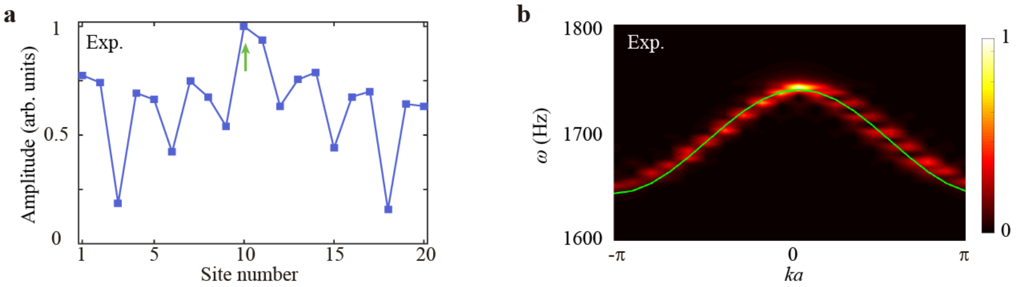

Supplementary Fig. 8 Experimental results of the reciprocal acoustic crystal. a Field distribution at frequency

ω = 1713 Hz. **b** Measured dispersion. The green line denotes the numerically fitted dispersion.

Supplementary Note 10: Measured field intensity distributions in the acoustic crystal with the next-nearest-neighbor non-reciprocal coupling

In this section, we have plotted Fig. S9 to show the measured field distributions at different frequencies for both winding numbers. One can see that below the frequency of the Bloch point (around 1696 Hz), the wave excited from site 10 is strongly suppressed towards the left boundary, but is dramatically amplified towards the right boundary, implying the winding number of v = -1. On the contrary, the phenomenon is reversed above the Bloch point, indicating the flip of the sign of the winding number.

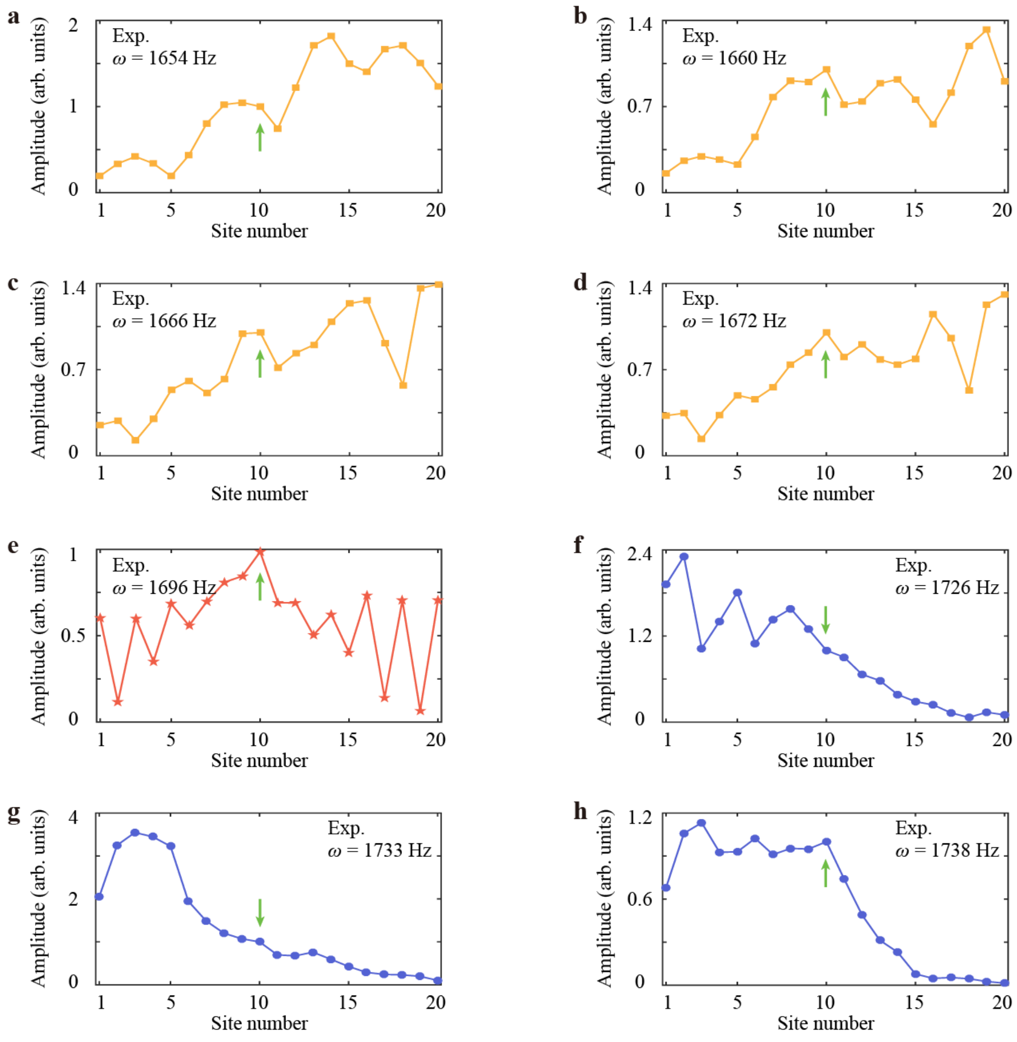

Supplementary Fig. 9 Measured field intensity distributions in the acoustic crystal with the next-nearest-neighbor non-reciprocal coupling. a-h Field intensity distributions at different frequencies. The measured results

are normalized by the amplitude at the source location (indicated by little arrows).

Supplementary Note 11: Theoretical calculation of field intensity distributions in the acoustic crystal with the next-nearest-neighbor non-reciprocal coupling

According to the coupled-mode theory, when the wave is incident to site 10 and detected at site 20, the dynamic equation can be described as,

$$da_1/dt = (-i\omega_0 - \gamma_0)a_1 - i\kappa_1 a_2 , \tag{S6}$$

$$da_2/dt = -i\kappa_1 a_1 + (-i\omega_0 - \gamma_0)a_2 - i\kappa_1 a_3 , \tag{S7}$$

$$da_3/dt = -i\tilde{\kappa}_a a_1 - i\kappa_1 a_2 + (-i\omega_0 - \gamma_0)a_3 - i\kappa_1 a_4 , \tag{S8}$$

$$\cdots$$

$$da_{10}/dt = -i\tilde{\kappa}_a a_8 - i\kappa_1 a_9 + (-i\omega_0 - \gamma_0 - \gamma_1)a_{10} - i\kappa_1 a_{11} + \sqrt{2\gamma_1} s_{1+} , \tag{S9}$$

$$\cdots$$

$$da_{19}/dt = -i\tilde{\kappa}_a a_{17} - i\kappa_1 a_{18} + (-i\omega_0 - \gamma_0)a_{19} - i\kappa_1 a_{20} , \tag{S10}$$

$$da_{20}/dt = -i\tilde{\kappa}_a a_{18} - i\kappa_1 a_{19} + (-i\omega_0 - \gamma_0 - \gamma_1)a_{20} , \tag{S11}$$

$$s_{2-} = \sqrt{2\gamma_1} a_{20} , \tag{S12}$$

$$s_{21} = s_{2-} / s_{1+} = \sqrt{2\gamma_1} a_{20} / s_{1+} , \tag{S13}$$

where a_n is the mode in site n, s_{1+} represents the incident wave from site 10, s_{2-} represents the detected output wave from site 20, and s_{21} represents the transmission coefficient. Similarly, the transmission can also be obtained when the wave is detected at each site. Then we plot the transmission versus the site number at different operating frequencies. As shown in Fig. S10, the theoretical results match well with the experimental counterparts (see Fig. 4e).

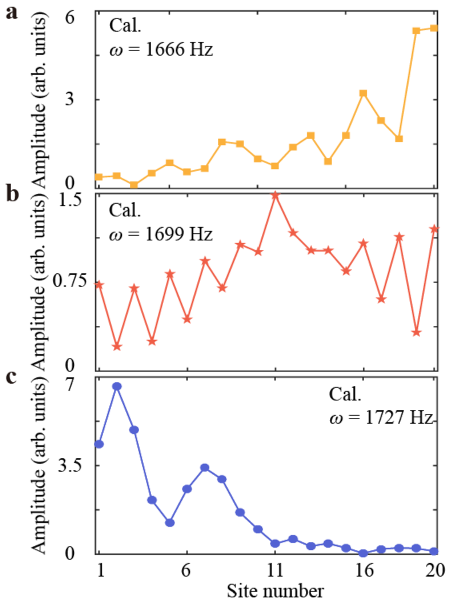

Supplementary Fig. 10 Calculated field intensity distributions in the acoustic crystal with the next-nearest-neighbor non-reciprocal coupling. a-c Calculated field intensity distributions in the acoustic crystal with the next-nearest-neighbor non-reciprocal coupling, when the source is placed at site 10 at frequency ω = 1666, 1699, and 1727 Hz, respectively. The measured results are normalized by the amplitude at site 10.

Supplementary Note 12: Transmission measurement of a pulse in the time domain in the acoustic crystal with the next-nearest-neighbor non-reciprocal coupling

In this section, we launch an acoustic pulse, covering the frequency range from 1600 to 1800 Hz, from site 1 (20) and measure the transmission at site 20 (1) in the time domain for the sample with the next-nearest-neighbor non-reciprocal coupling, as shown in Figs. S11(a) and 11(c). We also apply the Fourier transform to the signal to obtain the corresponding frequency spectra. Due to the twisted winding topology, the pulse is amplified towards the right boundary within frequency ranging from 1647 to 1675 Hz [see Fig. S11(b)]. On the contrary, the phenomenon is reversed when the pulse is launched at site 20, where the pulse is amplified towards the left boundary within frequency ranging from 1714 to 1736 Hz [see Fig. S11(d)].

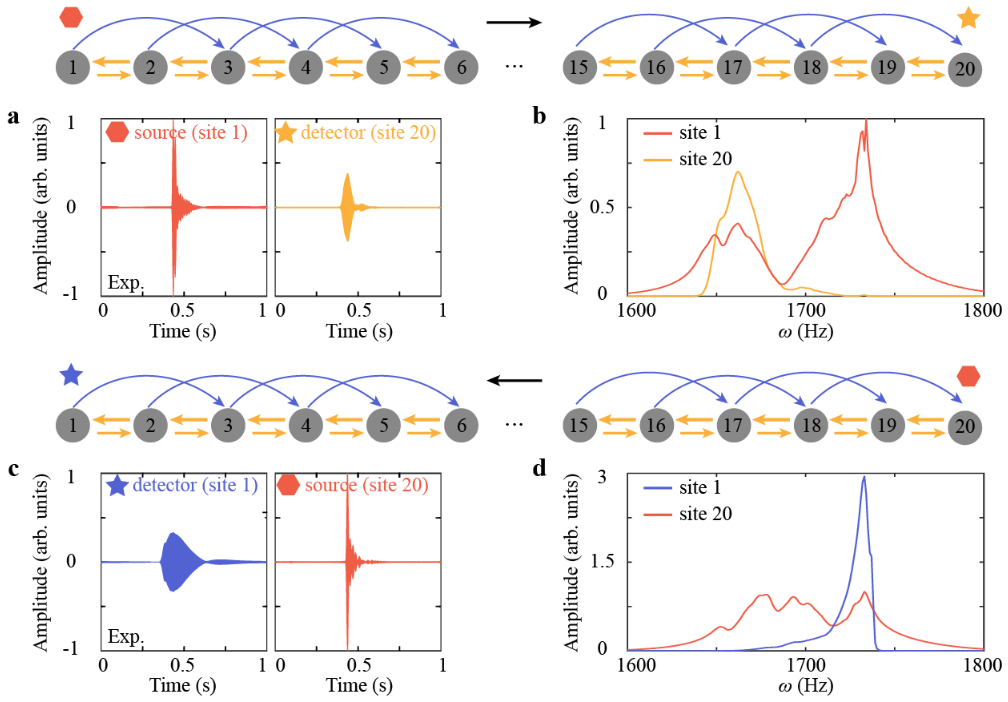

Supplementary Fig. 11 Transmission measurement of a pulse in the time domain in the acoustic crystal with the next-nearest-neighbor non-reciprocal coupling. a, **c** Transmission measurement of a pulse launched at site 1 (20) in the time domain with frequencies ranging from 1600 to 1800 Hz, respectively. The measured results are normalized by the amplitude at the source location. **b, d** Frequency spectra obtained by applying Fourier transform to the time-domain signal. The measured results are normalized by the amplitude at the source location. The top panel in each figure shows the schematic diagram with the source indicated by a hexagon and the detector marked by a star.

Supplementary Note 13: Measured field intensity distributions with different excitations in the acoustic crystal with the next-nearest-neighbor non-reciprocal coupling

For the acoustic crystal with the next-nearest-neighbor non-reciprocal coupling, we can still observe the field distribution localized at one boundary will exhibit stronger amplitude, when the source is placed further away from the boundary. Here, we place the source at the site 6, 8 and 10, and detect the response at each site of the chain, respectively. Below the Bloch point frequency (around 1690 Hz), the wave intensity at the right boundary increases as the excitation moves to the lower site number [see Fig. S12(a)]. Above the Bloch point frequency, the wave intensity at the left boundary increases as the excitation moves to the higher site number [see Fig. S12 (c)]. At the Bloch

point, the wave intensity at both boundaries is insensitive to the excitation position [see Fig. S12 (b)].

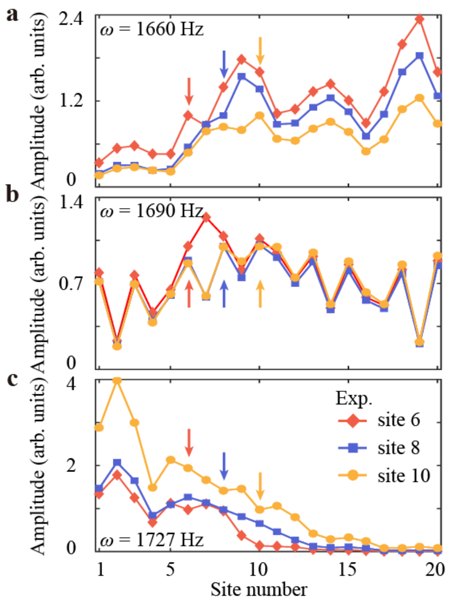

Supplementary Fig. 12 Measured field intensity distributions in the acoustic crystal with the next-nearest-neighbor non-reciprocal coupling. a-c Measured field intensity distribution at frequency ω = 1660, 1690, and 1727 Hz, when the source is located at the site 6, 8, and 10, respectively. The measured results are normalized by the amplitude at the source location (indicated by little arrows).

Supplementary Note 14: Tight-binding model for the coupled acoustic resonators

Due to the versatile tunability and ease of fabrication, the acoustic system is widely adapted for realizing tight-binding models. Here, we show an example to briefly introduce the connection between the designed acoustic structure and the tight-binding model. As depicted in Fig. S13(a), a single acoustic resonator has a dipole mode with eigenfrequency ω_0 = 1700 Hz. When two resonators are connected by narrow waveguides, the coupling between two dipole modes will lead to eigenfrequency splitting. The resulting symmetric (at 1685 Hz) and antisymmetric (at 1728 Hz) modes are shown in Fig. S13(b). Such a two-resonator system can be described by a two-level Hamiltonian $H = \begin{bmatrix} \omega_0 & \kappa \\ \kappa & \omega_0 \end{bmatrix}$, with κ being the coupling strength. Continuously increasing the number of resonators, the eigenfrequency splitting process will result in many eigenfrequencies

covering a certain frequency range. Eventually, the coupled resonators form a 1D infinite-size periodic chain that has a continuous band structure, which can be described by a Hamiltonian $H = \kappa_1 e^{ika} + \kappa_1 e^{-ika} + \omega_0$, with k being the wavevector and a being the lattice constant [see Fig. S13 (c)].

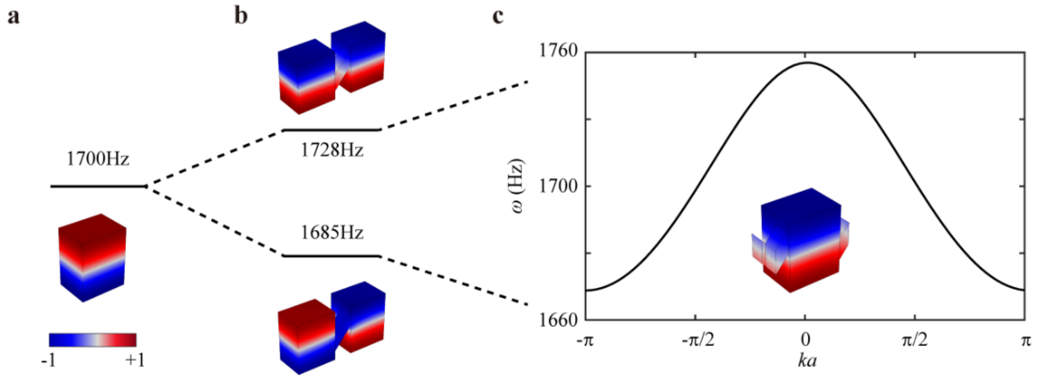

Supplementary Fig. 13 Tight-binding model for the coupled acoustic resonators. a Eigen frequency and eigen mode of a single acoustic resonator. **b** Eigen frequencies and eigen modes of two coupled resonators. **c** Band structure and an eigenmode of a 1D chain consisting of coupled resonators.

Supplementary Note 15: Topological robustness of the non-Hermitian skin effect (NHSE)

To test the robustness of the NHSE, we introduce a uniformly distributed disorder $\sigma_\kappa \in [-d_\kappa, d_\kappa]$ ($\sigma_\varphi \in [-d_\varphi, d_\varphi]$) to the amplitude κ_a (phase φ) of the unidirectional coupling $i\kappa_a e^{i\varphi}$. We calculate the first spatial moment of every eigenstate ψ_n, which is defined as[3],

$$m_1 = \sum_{n=0}^{n_{max}} n |\psi_n|^2 , \tag{S14}$$

with n being the position of the site. For each set of the first spatial moment, we calculate the averaged standard deviation σ_{m1}. The lattice size is $N = 100$, and each calculation is averaged over 1000 realizations. As shown in Fig. S14(a) and Fig. S15(a), the averaged standard deviation starts from 0 and converges at the large disorder strength, indicating the transition from the NHSE to the Anderson localization. We also show the eigenstates at different disorder strengths [see Figs. S14(b)-14(d) and S15(b)-15(d)]. One can see that at small disorder strength, the NHSE survives[3].

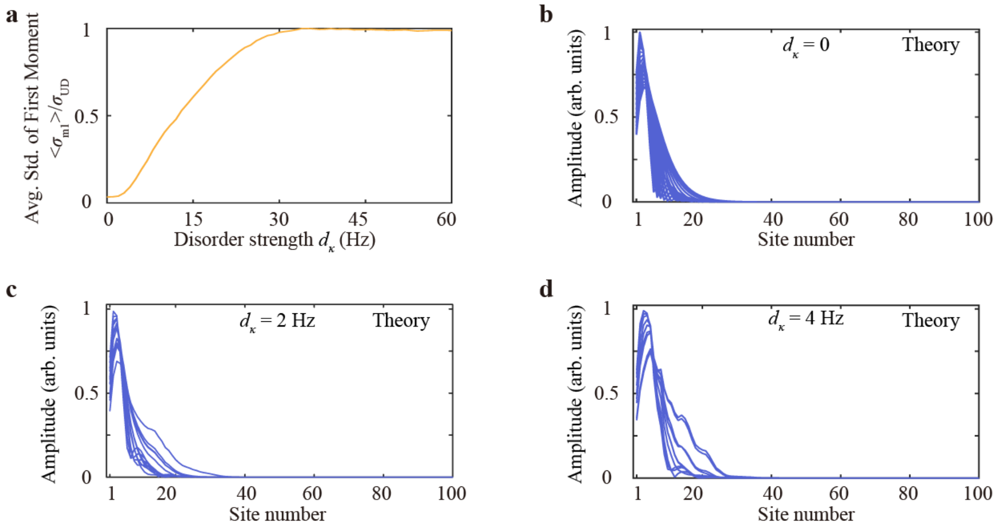

Supplementary Fig. 14 Robustness of the NHSE. a The averaged standard deviation of the first spatial moment versus the disorder strength d_κ. **b-d** Superposition of analytically-calculated field distributions of eigenstates at a disorder strength of 0 Hz (**b**), 2 Hz (**c**), and 4 Hz (**d**), respectively.

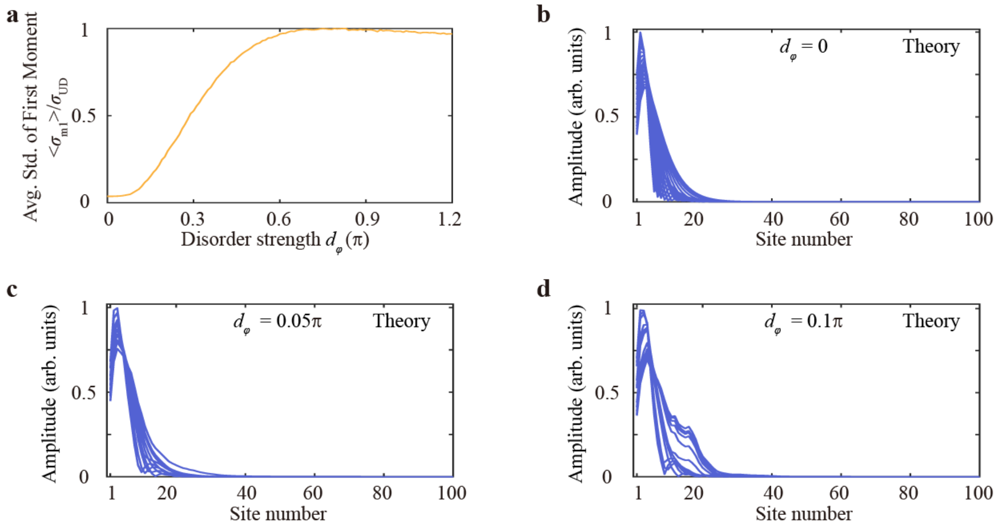

Supplementary Fig. 15 Robustness of the NHSE. a The averaged standard deviation of the first spatial moment versus the disorder strength d_φ. **b-d** Superposition of analytically-calculated field distributions of eigenstates at a disorder strength of 0 (**b**), 0.05π (**c**), and 0.1π (**d**), respectively.

Supplementary Note 16: Robustness of the Bloch-wave-like extended eigenstate

The Bloch-wave-like extended eigenstate at the Bloch point is also robust against moderate

disorder. To test its robustness, we introduce a uniformly distributed disorder $\sigma_\kappa \in [-d_\kappa, d_\kappa]$ ($\sigma_\varphi \in [-d_\varphi, d_\varphi]$) to the amplitude κ_a (phase φ) of the unidirectional coupling $i\kappa_a e^{i\varphi}$. The lattice size is N =100, and the model has reciprocal nearest-neighbor coupling κ_1 and unidirectional next-nearest-neighbor coupling $\tilde{\kappa}_a$. We plot all the eigenstates as a function of eigenfrequencies[4]. As shown in Fig. S16 and S17, most eigenstates are localized at the left or right boundary, and a Bloch-wave-like extended eigenstate exists at the frequency between the left and right localized states.

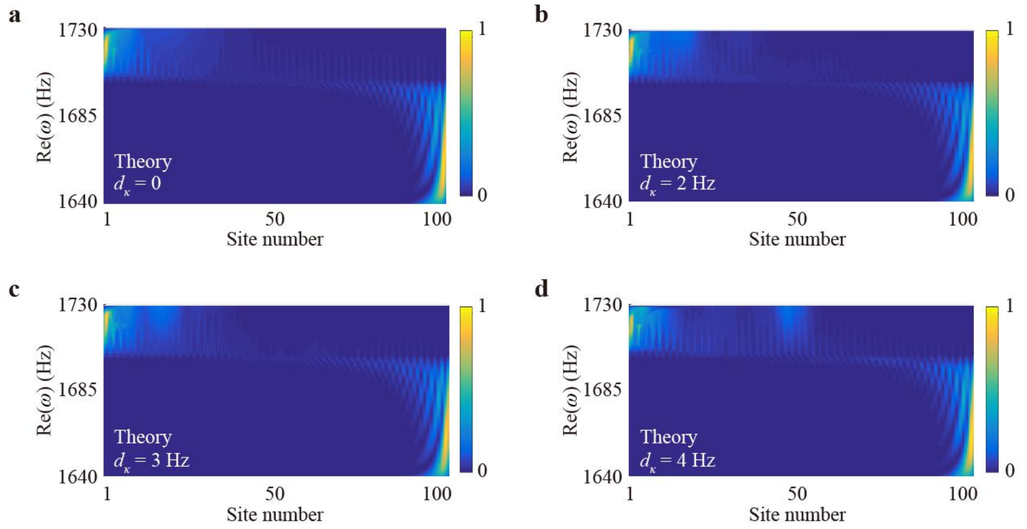

Supplementary Fig. 16 Robustness of the Bloch-wave-like extended eigenstate. a-d Analytically calculated energy of all eigenstates plotted as a function of eigenfrequencies and site number at different disorder strengths σ_κ of 0 Hz (**a**), 2 Hz (**b**), 3 Hz (**c**), and 4 Hz (**d**), respectively. Eigenstate profiles are normalized to have a maximum value 1.

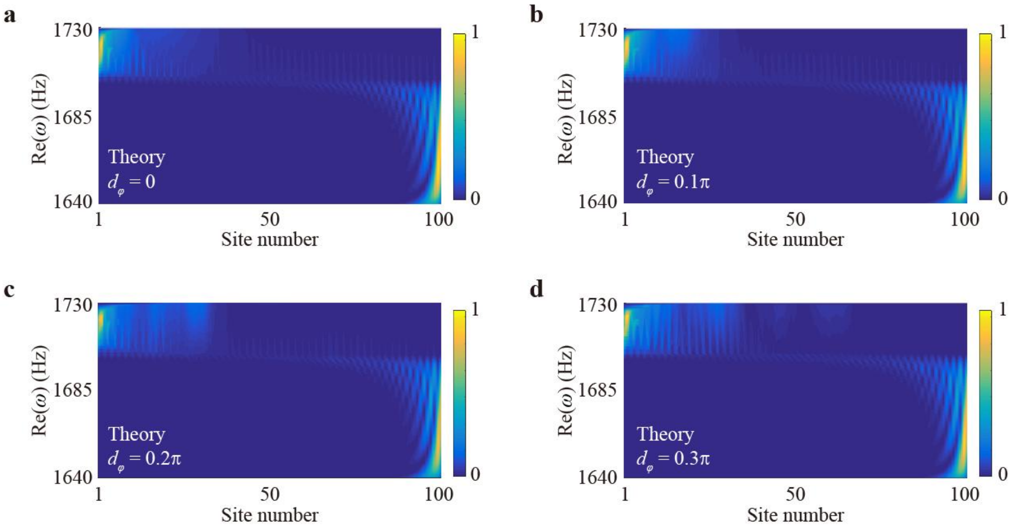

Supplementary Fig. 17 Robustness of the Bloch-wave-like extended eigenstate. a-d Analytically calculated energy of all eigenstates plotted as a function of eigenfrequencies and site number at different disorder strengths σ_φ of 0 (**a**), 0.1π (**b**), 0.2π (**c**), and 0.3π (**d**), respectively. Eigenstate profiles are normalized to have a maximum value 1.

References

1. Haus, H. *Waves and Fields in Optoelectronics* (Pretice Hall, 1984).
2. Helbig, T. et al. Generalized bulk–boundary correspondence in non-Hermitian topolectrical circuits. *Nat. Phys.* **16**, 747-750 (2020).
3. Weidemann, S. *et al.* Topological funneling of light. *Science* **368**, 311-314 (2020).
4. Song, F., Yao, S. & Wang, Z. Non-Hermitian topological invariants in real space. *Phys. Rev. Lett.* **123**, 246801 (2019).
